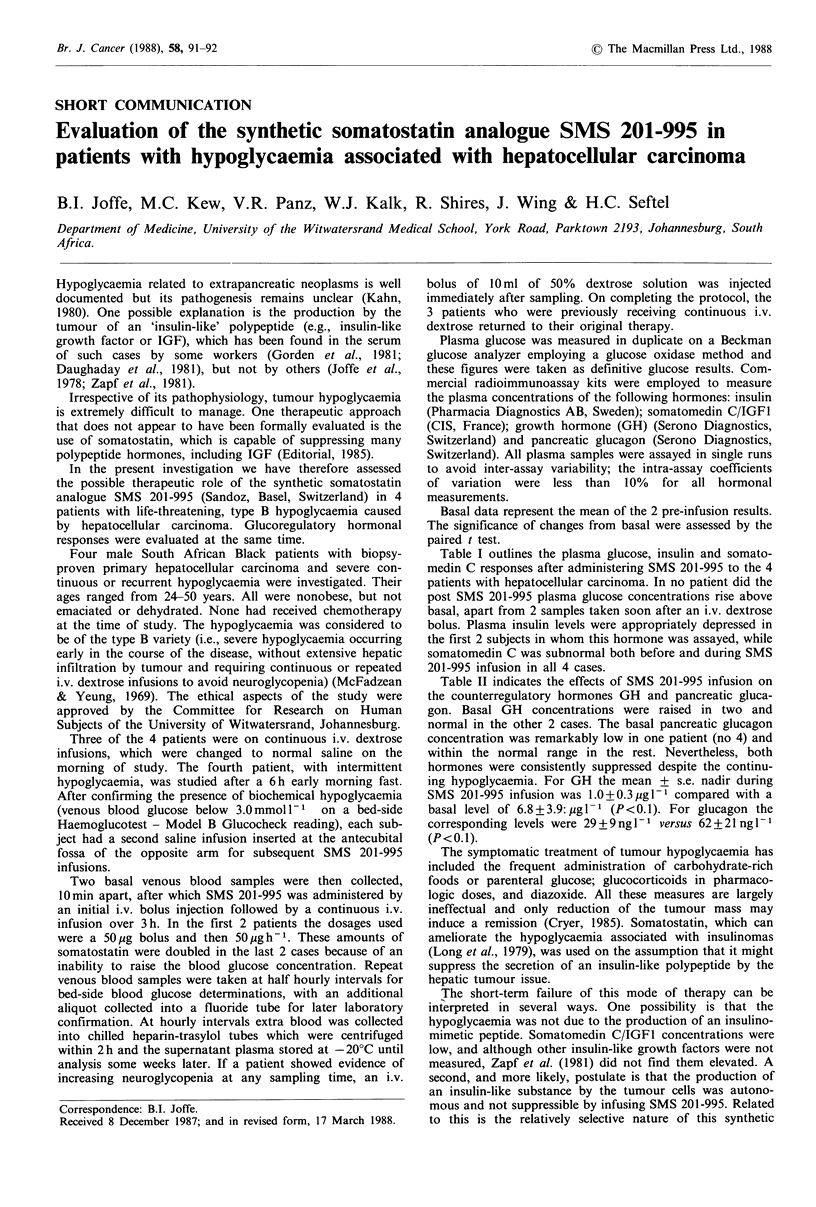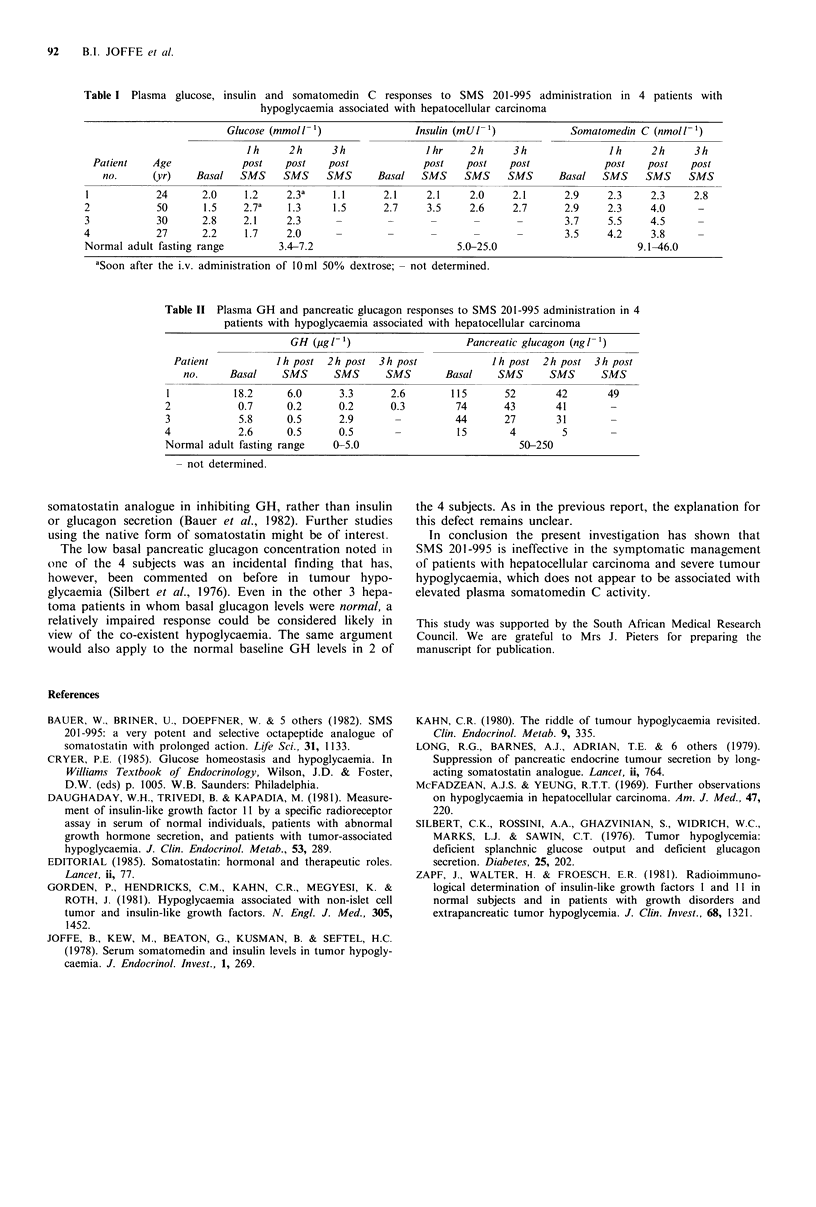# Evaluation of the synthetic somatostatin analogue SMS 201-995 in patients with hypoglycaemia associated with hepatocellular carcinoma.

**DOI:** 10.1038/bjc.1988.170

**Published:** 1988-07

**Authors:** B. I. Joffe, M. C. Kew, V. R. Panz, W. J. Kalk, R. Shires, J. Wing, H. C. Seftel

**Affiliations:** Department of Medicine, University of the Witwatersrand Medical School, Parktown, Johannesburg, South Africa.


*Br. J. Cancer* (1988), **58**, 91–92 © The Macmillan Press Ltd., 1988

**SHORT COMMUNICATION**

### Evaluation of the synthetic somatostatin analogue SMS 201-995 in patients with hypoglycaemia associated with hepatocellular carcinoma

B.I. Joffe, M.C. Kew, V.R. Panz, W.J. Kalk, R. Shires, J. Wing & H.C. Seftel

*Department of Medicine, University of the Witwatersrand Medical School, York Road, Parktown 2193, Johannesburg, South Africa.*

Hypoglycaemia related to extrapancreatic neoplasms is well documented but its pathogenesis remains unclear (Kahn, 1980). One possible explanation is the production by the tumour of an 'insulin-like' polypeptide (e.g., insulin-like growth factor or IGF), which has been found in the serum of such cases by some workers (Gorden *et al.*, 1981; Daughaday *et al.*, 1981), but not by others (Joffe *et al.*, 1978; Zapf *et al.*, 1981).

Irrespective of its pathophysiology, tumour hypoglycaemia is extremely difficult to manage. One therapeutic approach that does not appear to have been formally evaluated is the use of somatostatin, which is capable of suppressing many polypeptide hormones, including IGF (Editorial, 1985).

In the present investigation we have therefore assessed the possible therapeutic role of the synthetic somatostatin analogue SMS 201-995 (Sandoz, Basel, Switzerland) in 4 patients with life-threatening, type B hypoglycaemia caused by hepatocellular carcinoma. Glucoregulatory hormonal responses were evaluated at the same time.

Four male South African Black patients with biopsy-proven primary hepatocellular carcinoma and severe continuous or recurrent hypoglycaemia were investigated. Their ages ranged from 24–50 years. All were nonobese, but not emaciated or dehydrated. None had received chemotherapy at the time of study. The hypoglycaemia was considered to be of the type B variety (i.e., severe hypoglycaemia occurring early in the course of the disease, without extensive hepatic infiltration by tumour and requiring continuous or repeated i.v. dextrose infusions to avoid neuroglycopenia) (McFadzean & Yeung, 1969). The ethical aspects of the study were approved by the Committee for Research on Human Subjects of the University of Witwatersrand, Johannesburg.

Three of the 4 patients were on continuous i.v. dextrose infusions, which were changed to normal saline on the morning of study. The fourth patient, with intermittent hypoglycaemia, was studied after a 6 h early morning fast. After confirming the presence of biochemical hypoglycaemia (venous blood glucose below $3.0\,\mathrm{mmol\,l^{-1}}$ on a bed-side Haemoglucotest – Model B Glucocheck reading), each subject had a second saline infusion inserted at the antecubital fossa of the opposite arm for subsequent SMS 201-995 infusions.

Two basal venous blood samples were then collected, 10 min apart, after which SMS 201-995 was administered by an initial i.v. bolus injection followed by a continuous i.v. infusion over 3 h. In the first 2 patients the dosages used were a $50\,\mu\mathrm{g}$ bolus and then $50\,\mu\mathrm{g\,h^{-1}}$. These amounts of somatostatin were doubled in the last 2 cases because of an inability to raise the blood glucose concentration. Repeat venous blood samples were taken at half hourly intervals for bed-side blood glucose determinations, with an additional aliquot collected into a fluoride tube for later laboratory confirmation. At hourly intervals extra blood was collected into chilled heparin-trasylol tubes which were centrifuged within 2 h and the supernatant plasma stored at $-20^{\circ}\mathrm{C}$ until analysis some weeks later. If a patient showed evidence of increasing neuroglycopenia at any sampling time, an i.v. bolus of 10 ml of 50% dextrose solution was injected immediately after sampling. On completing the protocol, the 3 patients who were previously receiving continuous i.v. dextrose returned to their original therapy.

Plasma glucose was measured in duplicate on a Beckman glucose analyzer employing a glucose oxidase method and these figures were taken as definitive glucose results. Commercial radioimmunoassay kits were employed to measure the plasma concentrations of the following hormones: insulin (Pharmacia Diagnostics AB, Sweden); somatomedin C/IGF1 (CIS, France); growth hormone (GH) (Serono Diagnostics, Switzerland) and pancreatic glucagon (Serono Diagnostics, Switzerland). All plasma samples were assayed in single runs to avoid inter-assay variability; the intra-assay coefficients of variation were less than 10% for all hormonal measurements.

Basal data represent the mean of the 2 pre-infusion results. The significance of changes from basal were assessed by the paired *t* test.

Table I outlines the plasma glucose, insulin and somatomedin C responses after administering SMS 201-995 to the 4 patients with hepatocellular carcinoma. In no patient did the post SMS 201-995 plasma glucose concentrations rise above basal, apart from 2 samples taken soon after an i.v. dextrose bolus. Plasma insulin levels were appropriately depressed in the first 2 subjects in whom this hormone was assayed, while somatomedin C was subnormal both before and during SMS 201-995 infusion in all 4 cases.

Table II indicates the effects of SMS 201-995 infusion on the counterregulatory hormones GH and pancreatic glucagon. Basal GH concentrations were raised in two and normal in the other 2 cases. The basal pancreatic glucagon concentration was remarkably low in one patient (no 4) and within the normal range in the rest. Nevertheless, both hormones were consistently suppressed despite the continuing hypoglycaemia. For GH the mean $\pm$ s.e. nadir during SMS 201-995 infusion was $1.0\pm0.3\,\mu\mathrm{g\,l^{-1}}$ compared with a basal level of $6.8\pm3.9\!:\mu\mathrm{g\,l^{-1}}$ ($P<0.1$). For glucagon the corresponding levels were $29\pm9\,\mathrm{ng\,l^{-1}}$ *versus* $62\pm21\,\mathrm{ng\,l^{-1}}$ ($P<0.1$).

The symptomatic treatment of tumour hypoglycaemia has included the frequent administration of carbohydrate-rich foods or parenteral glucose; glucocorticoids in pharmacologic doses, and diazoxide. All these measures are largely ineffectual and only reduction of the tumour mass may induce a remission (Cryer, 1985). Somatostatin, which can ameliorate the hypoglycaemia associated with insulinomas (Long *et al.*, 1979), was used on the assumption that it might suppress the secretion of an insulin-like polypeptide by the hepatic tumour issue.

The short-term failure of this mode of therapy can be interpreted in several ways. One possibility is that the hypoglycaemia was not due to the production of an insulinomimetic peptide. Somatomedin C/IGF1 concentrations were low, and although other insulin-like growth factors were not measured, Zapf *et al.* (1981) did not find them elevated. A second, and more likely, postulate is that the production of an insulin-like substance by the tumour cells was autonomous and not suppressible by infusing SMS 201-995. Related to this is the relatively selective nature of this synthetic

Correspondence: B.I. Joffe.
Received 8 December 1987; and in revised form, 17 March 1988.

**Table I** Plasma glucose, insulin and somatomedin C responses to SMS 201-995 administration in 4 patients with hypoglycaemia associated with hepatocellular carcinoma

| | | Glucose ($mmol\,l^{-1}$) | | | | Insulin ($mU\,l^{-1}$) | | | | Somatomedin C ($nmol\,l^{-1}$) | | | |
|---|---|---|---|---|---|---|---|---|---|---|---|---|---|
| Patient no. | Age (yr) | Basal | 1 h post SMS | 2 h post SMS | 3 h post SMS | Basal | 1 hr post SMS | 2 h post SMS | 3 h post SMS | Basal | 1 h post SMS | 2 h post SMS | 3 h post SMS |
| 1 | 24 | 2.0 | 1.2 | 2.3[a] | 1.1 | 2.1 | 2.1 | 2.0 | 2.1 | 2.9 | 2.3 | 2.3 | 2.8 |
| 2 | 50 | 1.5 | 2.7[a] | 1.3 | 1.5 | 2.7 | 3.5 | 2.6 | 2.7 | 2.9 | 2.3 | 4.0 | – |
| 3 | 30 | 2.8 | 2.1 | 2.3 | – | – | – | – | – | 3.7 | 5.5 | 4.5 | – |
| 4 | 27 | 2.2 | 1.7 | 2.0 | – | – | – | – | – | 3.5 | 4.2 | 3.8 | – |
| Normal adult fasting range | | | | 3.4–7.2 | | | | 5.0–25.0 | | | | 9.1–46.0 | |

[a]Soon after the i.v. administration of 10 ml 50% dextrose; – not determined.

**Table II** Plasma GH and pancreatic glucagon responses to SMS 201-995 administration in 4 patients with hypoglycaemia associated with hepatocellular carcinoma

| | GH ($\mu g\,l^{-1}$) | | | | Pancreatic glucagon ($ng\,l^{-1}$) | | | |
|---|---|---|---|---|---|---|---|---|
| Patient no. | Basal | 1 h post SMS | 2 h post SMS | 3 h post SMS | Basal | 1 h post SMS | 2 h post SMS | 3 h post SMS |
| 1 | 18.2 | 6.0 | 3.3 | 2.6 | 115 | 52 | 42 | 49 |
| 2 | 0.7 | 0.2 | 0.2 | 0.3 | 74 | 43 | 41 | – |
| 3 | 5.8 | 0.5 | 2.9 | – | 44 | 27 | 31 | – |
| 4 | 2.6 | 0.5 | 0.5 | – | 15 | 4 | 5 | – |
| Normal adult fasting range | | | 0–5.0 | | | 50–250 | | |

– not determined.

somatostatin analogue in inhibiting GH, rather than insulin or glucagon secretion (Bauer *et al.*, 1982). Further studies using the native form of somatostatin might be of interest.

The low basal pancreatic glucagon concentration noted in one of the 4 subjects was an incidental finding that has, however, been commented on before in tumour hypoglycaemia (Silbert *et al.*, 1976). Even in the other 3 hepatoma patients in whom basal glucagon levels were *normal*, a relatively impaired response could be considered likely in view of the co-existent hypoglycaemia. The same argument would also apply to the normal baseline GH levels in 2 of the 4 subjects. As in the previous report, the explanation for this defect remains unclear.

In conclusion the present investigation has shown that SMS 201-995 is ineffective in the symptomatic management of patients with hepatocellular carcinoma and severe tumour hypoglycaemia, which does not appear to be associated with elevated plasma somatomedin C activity.

This study was supported by the South African Medical Research Council. We are grateful to Mrs J. Pieters for preparing the manuscript for publication.

### References

BAUER, W., BRINER, U., DOEPFNER, W. & 5 others (1982). SMS 201-995: a very potent and selective octapeptide analogue of somatostatin with prolonged action. *Life Sci.*, **31**, 1133.

CRYER, P.E. (1985). Glucose homeostasis and hypoglycaemia. In *Williams Textbook of Endocrinology*, Wilson, J.D. & Foster, D.W. (eds) p. 1005. W.B. Saunders: Philadelphia.

DAUGHADAY, W.H., TRIVEDI, B. & KAPADIA, M. (1981). Measurement of insulin-like growth factor 11 by a specific radioreceptor assay in serum of normal individuals, patients with abnormal growth hormone secretion, and patients with tumor-associated hypoglycaemia. *J. Clin. Endocrinol. Metab.*, **53**, 289.

EDITORIAL (1985). Somatostatin: hormonal and therapeutic roles. *Lancet*, **ii**, 77.

GORDEN, P., HENDRICKS, C.M., KAHN, C.R., MEGYESI, K. & ROTH, J. (1981). Hypoglycaemia associated with non-islet cell tumor and insulin-like growth factors. *N. Engl. J. Med.*, **305**, 1452.

JOFFE, B., KEW, M., BEATON, G., KUSMAN, B. & SEFTEL, H.C. (1978). Serum somatomedin and insulin levels in tumor hypoglycaemia. *J. Endocrinol. Invest.*, **1**, 269.

KAHN, C.R. (1980). The riddle of tumour hypoglycaemia revisited. *Clin. Endocrinol. Metab.* **9**, 335.

LONG, R.G., BARNES, A.J., ADRIAN, T.E. & 6 others (1979). Suppression of pancreatic endocrine tumour secretion by long-acting somatostatin analogue. *Lancet*, **ii**, 764.

McFADZEAN, A.J.S. & YEUNG, R.T.T. (1969). Further observations on hypoglycaemia in hepatocellular carcinoma. *Am. J. Med.*, **47**, 220.

SILBERT, C.K., ROSSINI, A.A., GHAZVINIAN, S., WIDRICH, W.C., MARKS, L.J. & SAWIN, C.T. (1976). Tumor hypoglycemia: deficient splanchnic glucose output and deficient glucagon secretion. *Diabetes*, **25**, 202.

ZAPF, J., WALTER, H. & FROESCH, E.R. (1981). Radioimmunological determination of insulin-like growth factors 1 and 11 in normal subjects and in patients with growth disorders and extrapancreatic tumor hypoglycemia. *J. Clin. Invest.*, **68**, 1321.